# Enlarged fistulotomy of the papilla as access to the biliary tract during ERCP

**DOI:** 10.1186/s12876-023-03013-w

**Published:** 2023-11-29

**Authors:** Alexandre Gomes, Ana Sarah Rafka Haidar, Giovani Caetano Padilha, Juliana Bara, Mariana Sussai Nonato, José Mauro da Silva Rodrigues, Pérsio Campos Correia Pinto, Ricardo de Oliveira Ayres, Ronaldo Antonio Borghesi

**Affiliations:** Department of Surgery, Faculty of Medical Sciences and Health, Pontifical Catholic University of São Paulo (FCMB / PUC-SP), São Paulo, Brazil

**Keywords:** ERCP, Catheterization, Endoscopic sphincterotomy

## Abstract

**Background:**

Demonstration of access to the bile duct through Enlarged Papillary Fistulotomy, a method different from conventional fistulotomy.

**Aims:**

Demonstration of the EFP technique with dissection in layers of the papilla for accessing the common bile duct, its efficiency and safety, rescue of cases of failure in cannulation and cases of access failure by EFP in the first attempt, facilitating cannulation in the second attempt.

**Methods:**

Cross-sectional study, with retrospective data collection from 2233 ERCP exams with 528 EFP procedures, analysis of success and complications.

**Results:**

528 patients underwent EFP on the first attempt, with success in 465 cases (88.06%) and 63 failures (11.94%). Of these failures, 33 patients (52.38%) returned for a second EFP attempt, with success in 30 cases (90.9%) and failure in 3 cases (9.1%). Deep bile duct cannulation was achieved in 93.75% of EFP procedures, and cannulation failure occurred in 33 cases (6.25%).

**Conclusion:**

EFP showed efficiency in CBD cannulation, did not induce post-ERCP pancreatitis, no cases of perforation or false tract, but resulted in higher rates of minor bleeding, rescued cases of access failure by EFP, facilitated the posterior approach on the second attempt, it is safe, effective, low risk and associated with few comorbidities.

**Supplementary Information:**

The online version contains supplementary material available at 10.1186/s12876-023-03013-w.

## Introduction

ERCP is an important therapeutic method for clearing the extrahepatic bile duct [1]. Access to the common bile duct is critical for the endoscopic treatment of biliary obstruction. Cannulation through the papillary ostium (CTPO) is not always possible and cannulation failure occurs in 18% of cases [2]. The insistence on attempting cannulation through the ostium, with numerous attempts at progression of the guide wire and the injection of contrast into the pancreatic duct is responsible for most cases of pancreatitis [3, 4].

Thus, avoiding excessive trauma to the papillary ostium and biliopancreatic junction in cannulation attempts is of fundamental importance to avoid acute pancreatitis [5]. We have been using early fistulotomy with EFP in all cases since 2006, with the technique detailed ahead. With this approach we observed that the success rate of deep bile duct cannulation improved and the cases of post-ERCP pancreatitis decreased. Han SY (2021) showed that the rate of post-ERCP pancreatitis was significantly affected by the level of endoscopist experience in patients who underwent cannulation and CP, but there was no difference when the technique used was fistulotomy [6].

It is well established that cannulation should be performed with a sphincterotome and guide wire and not with a simple catheter [7]. Guidewire use increases cannulation success (97% vs. 67% single catheter), facilitates selective biliary cannulation, limits papilla trauma, decreases inadvertent pancreatic duct contrast injection, and decreases the risk of post-ERCP pancreatitis [2]. The 0.025 and 0.035 wires do not appear to affect the success rate of selective cannulation or the incidence of pancreatitis [8]. Cannulation can be performed with the externalization of the guide wire from approximately half to 1 cm without the sphincterotome touching the papilla (no touch) or slight insertion of the sphincterotome and then advancement of the guide wire (touch) [9]. Failure of the guide wire to progress may be due to the shape of the distal common bile duct in the form of a sigmoid or siphon. One option is to inject a small amount of contrast through the ostium to outline the anatomy (contrast-assisted) with the use of a guide wire, which is the mixed technique. The maneuver of inserting the sphincterotome over the guide wire to its tip and then releasing the commands to rectify the S can facilitate guide wire entry [7].

Several factors can influence the success of cannulation: first, correct positioning, with the tip of the device located below the papila, and second, the size and appearance of the papilla. Small or pleated papillae are independent risk factors for difficult and time-consuming cannulation in fistulotomies. There are several anatomical variations that occur in the periampullary region. A small papilla has the highest chance of complications (12% failure, 52% cannulation difficulties, and 20% pancreatitis). Papillae with a common canal represent more than 75% of the cases, being more than 1.5 mm in the Y shape, which corresponds to 61.2% of the cases, less than 1.5 mm (in the V shape - 14.3% of the cases), without a common channel with an ostium (U-shaped - 22.4% of the cases) and in a separate ostia in 2.1% of the cases [10]. Peridiverticular or intradiverticular papillae may cause difficulties due to axis deviation. Some maneuvers, such as changing the position of the device, insertion of a long scope or pulling of the papilla with biopsy forceps, need to be performed for cannulation.

The standard technique fails to cannulate the bile duct in approximately 11% (range: 5 to 35%) of cases [10]. There are several techniques and maneuvers that can be used to gain access to the bile duct in case of failure in conventional cannulation. As alternatives, we have pre-cut techniques, the use of two guide wires, the use of pancreatic stent, and fistulotomy.

## Objectives

Herein, we describe the approach to accessing the bile duct through the Enlarged Papillary Fistulotomy (EFP), which is a different method from conventional fistulotomy. This technique allows an approach with more visibility of the anatomical structures of the papilla, allowing easier and safer access. The wide dissection allows for an easier second attempt if the first approach fails, since the distal bile duct is exposed in the duodenal lumen. A bilioduodenal fistula forms, evidencing the output of bile from the fistular orifice and, allowing easier cannulation in the second approach. The complication and efficacy rates are reported.

## Methods

This is a cross-sectional study of patients who were referred for ERCP, routine and urgent cases, which were systematically evaluated and performed by operators with experience in the applied procedure.

Data collection was performed retrospectively from an electronic database, including exams performed from November 20, 2006 to August 12, 2022 at Endoclinic SP. A total of 2064 patients and 2233 consecutive ERCP exams were included in the initial analysis in this period.

Patients underwent ERCP examination with direct cannulation and CP (conventional papillotomy) or, if the latter failed, they migrated to the EFP group. All ERCP cases, both those submitted to CP and EFP, were singly evaluated, and both groups were analyzed in terms of cannulation success and its complications.

The evaluated patients underwent the examination and the diagnoses were grouped as follows: a) choledocholithiasis; b) ERCP with minimal changes, such as dilatation of the CBD beyond 12 mm in diameter without an obstructive factor, history of jaundice or pancreatitis, tests indicated by imaging or laboratory tests with unconfirmed suspicion of stones or obstruction. Obstructive bile duct neoplasms were divided and grouped as follows: c) pancreatic head neoplasm; d) neoplasms of the hepatic hilum (cholangiocarcinoma, extrinsic compression by metastases); and e) neoplasms of the papilla of Vater. Benign lesions were grouped into: f) benign fibrotic strictures (undetermined strictures, late postoperative sequelae, papillitis or fibrotic thinning of the distal common bile duct); g) Mirizzi syndrome; h) early postoperative complications of the bile ducts, such as partial or total ligations, fistulas, and strictures; i) sclerosing cholangitis; j) chronic pancreatitis; and k) other diagnoses, such as choledochal cyst, and cholelithiasis.

The exam reports and images obtained during this period were recorded and saved in a database (OCRAM® system). The data mined for the composition of the research were extracted from the relational database MySQL Community, version 5.5.40, software, entitled OCRAM Capture of Medical Images and Reports. This system was developed using the Java programming language and was used to capture the photos of the ERCP exams and compose the respective reports during the study period. The ERCP reports were typed using OCRAM software were structured in XML (Extensible Markup Language) format and followed an XML-Schema according to W3C (Worldwide Web Consortium) standards, resulting in a well-formed, valid and standardized structure of the ERCP reports in XML, this allowed the mining of terms referring to diseases to be performed reliably through the declarative search language SQL (Structured Query Language) in conjunction with an XML DOM (Document Object Model) parser [11].

### Inclusion criteria

All patients were submitted to an attempt at cannulation through the ostium of the papilla with a sphincterotome and guide wire. When direct cannulation of the common bile duct is not achieved and the guide wire goes into the pancreatic duct, we opt for the double guide wire technique. This technique was used in many of the cases described here and these cases were grouped as successful cannulation through the ostium. We defined cannulation failure after performing the following tactics: a) access to the common bile duct was not achieved after at least 4 attempts with a guide wire; b) attempts at cannulation after injection of a small amount of contrast to identify the common bile duct and the pancreatic duct; c) if the guidewire goes only to the pancreatic duct, we perform the double guidewire technique and, if the second guidewire fails to gain access to the biliary tract, we consider it as a failure. The cases of cannulation failure migrated to EFP, according to the criteria of the fistulotomy technique.

### Exclusion criteria

Of the 2064 patients initially evaluated in the electronic system, 105 patients in whom there were anatomical changes that made it impossible to perform ERCP: surgeries such as Roux-en-Y or Billroth II gastrectomy, or esophageal, stomach or duodenal stenosis were excluded. A total of 1959 patients and 2233 exams remained for the final analysis.

### Intervention

**Fig. 1:**
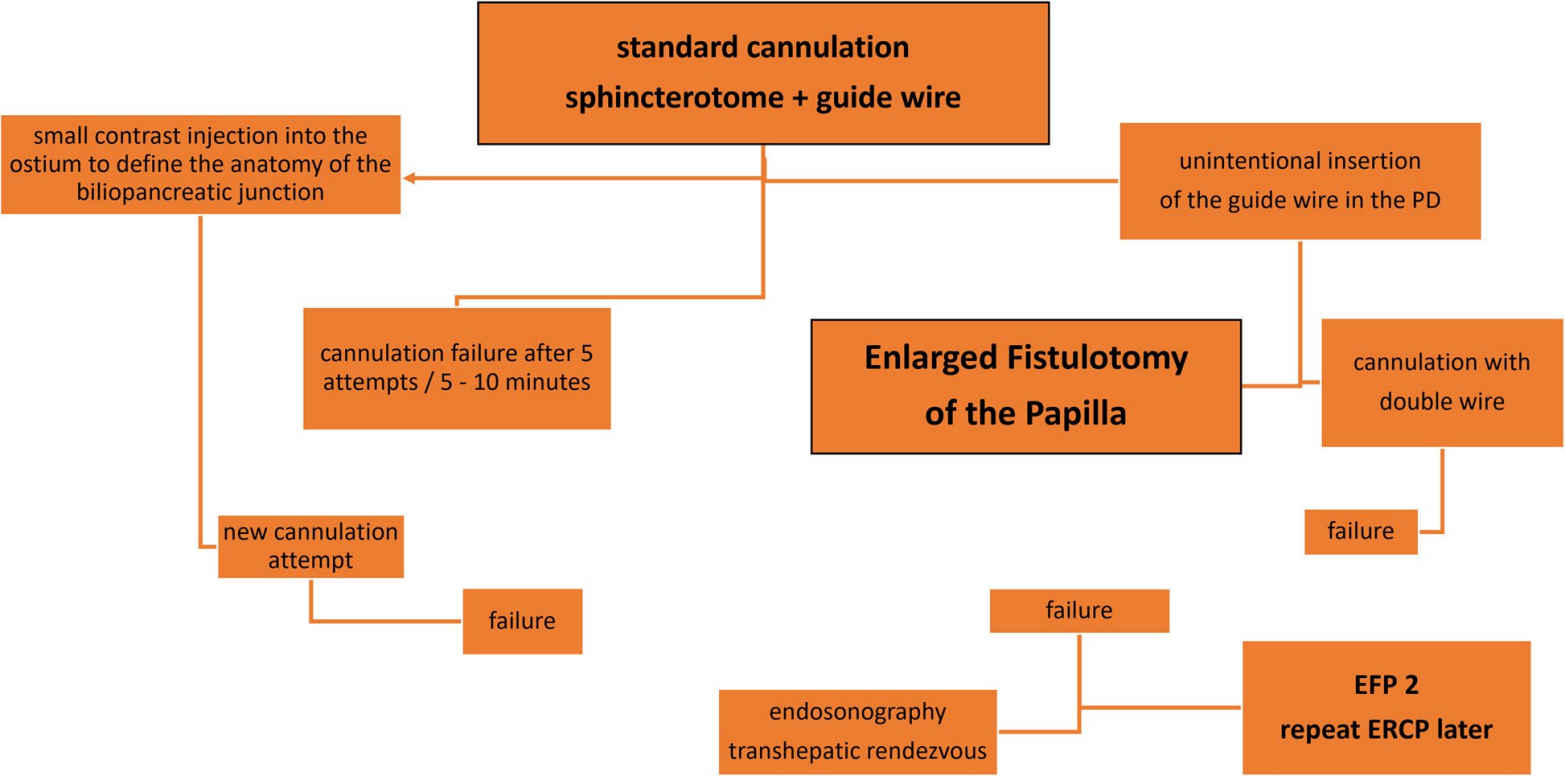
Algorithm for access to the common bile duct

All patients had their exams previously evaluated and underwent preparation with a 12-hour fast before the procedure. Patients who were using antiplatelet agents and anticoagulants were instructed to discontinue these medications. The use of ciprofloxacin 500 mg every 12 hours was indicated for all patients, starting from 6 hours when there was no increase in bilirubin and 24 hours previously in cases of bile duct obstruction in patients with elevated bilirubin levels. All patients were initially submitted to the standard cannulation technique, using the 3-way sphincterotome and 0.035 or 0.025 guide wire, depending on the availability of the brands Olympus®, Boston®, Cook®, MediGlobe®, Scitech® or GFE®. The WEM® electrosurgical generator, model SS200A, was used in all cases. In the case of cannulation failure, following the criteria for indicating early fistulotomy, the patients underwent EFP. After cannulation failure (defined as failing to introduce the guide wire into the bile duct five times, even after injecting a small amount of contrast into the papilla ostium, or the guide wire inadvertently being directed into the pancreatic duct), our preference was to perform EFP early, avoiding trauma to the papilla and the injection too much contrast (Fig. 1).

**Fig. 2:**
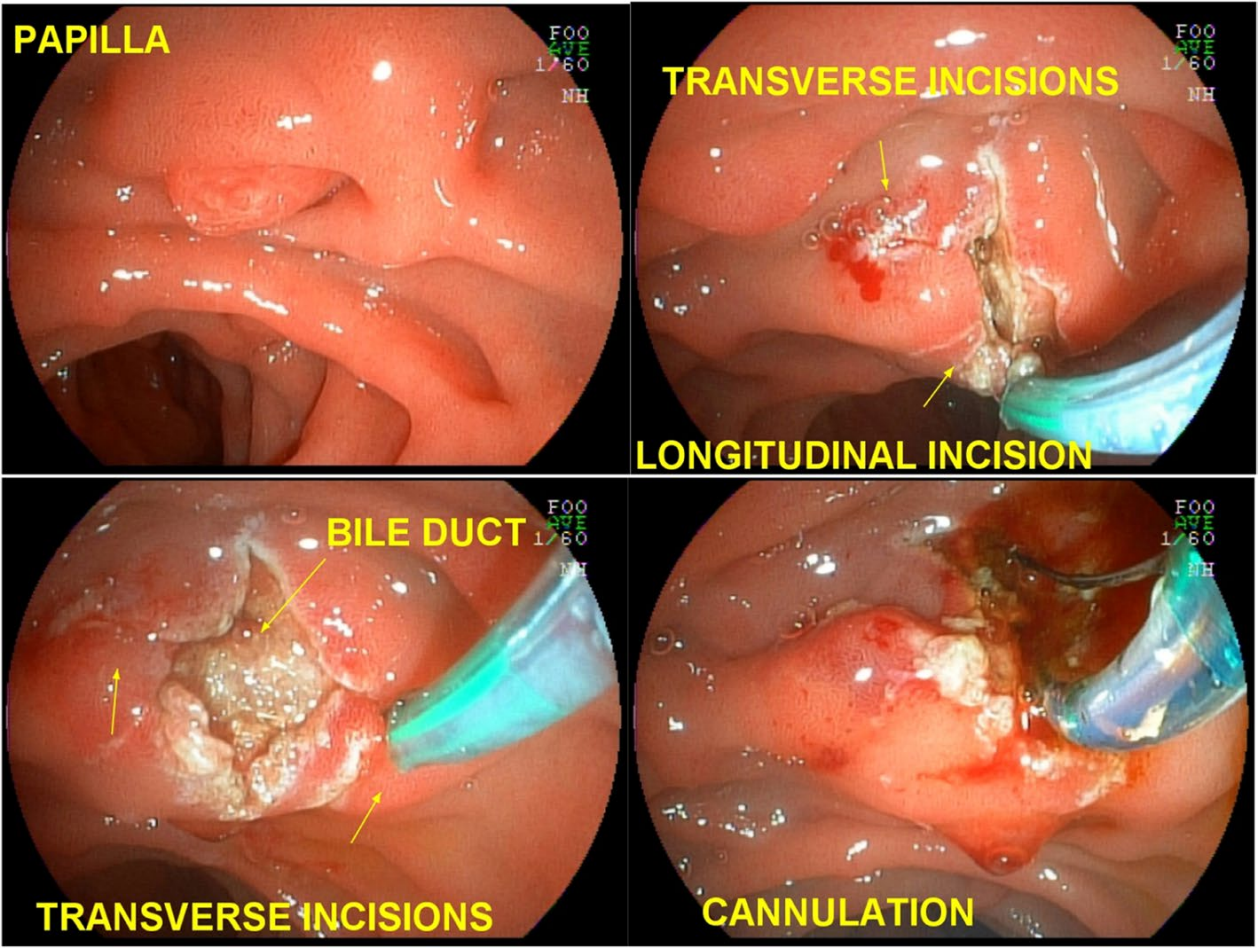
EFP with lateral incisions. **a** papilla of difficult cannulation with the guide wire; **b** wide longitudinal incision and lateral incisions; **c** exposure of the submucosa by pushing aside the mucosal edges, sectioning the musculature fibers until opening the common bile duct; **d** deep cannulation of the bile duct

After identification and “palpation” with the tip of the fistulotome of the lateral limits of the papilla and exposure of the infundibulum, a wide, shallow incision only of the papillary mucosa was iniciated and, purely cut, with the fistulotome needle adjusted to approximately 2 mm, from top to bottom, just below the transverse crease, avoiding opening the region of the common channel. With the needle-knife, the needle was retracted, and the mucosal edges were pushed aside to expose the submucosa. Lateral incisions were made to expand the exposure and then superficial incisions were made to open the submucosa and, dissect thin layers one at a time, interspersed by blunt dissection with the tip of the needle-knife retracted; these steps were followed by identification of vessels, hemostasis and exposure of the sphincter muscle of the distal common bile duct. If bleeding occured, washing was performed with pressurized water through the fistulotome catheter itself or through the working channel of the duodenoscope, the bleeding point was identified and captured with hot biopsy forceps and sealed by seizure, rapid hemostasis was achieved by coagulation, and underlying thermal tissue damage was avoided. At this stage, transversal accessory incisions were made to remove the mucosa over the papilla and improve exposure (Fig. 2).

**Fig. 3:**
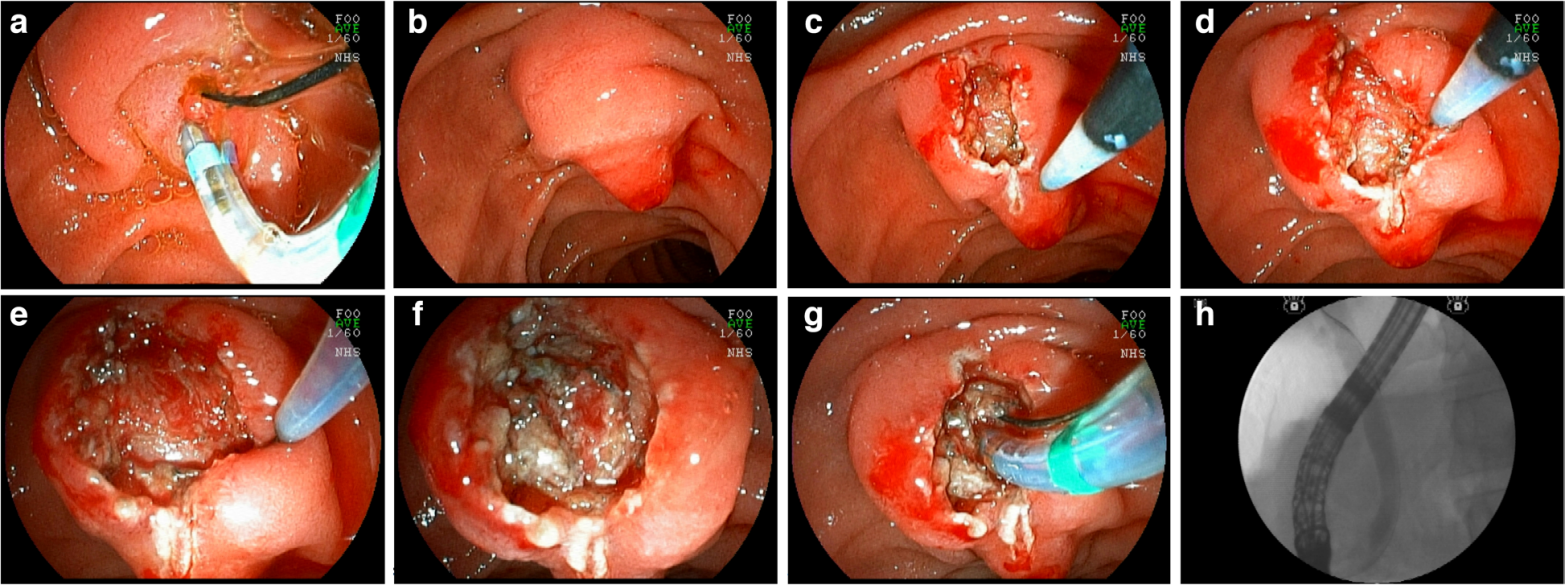
EFP with identification of the muscle tissue. **a** failed cannulation with the guide wire; **b** repositioning of the duodenoscope to visualize the infundibulum of the papilla; **c** Wide longitudinal incision from the transverse crease; **d** wide lateral incisions; **e** identification of the muscular sphincter of the papilla; **f** sectioning the muscular fibers to expose the common bile duct mucosa; **g** cannulation of the bile duct; **h** deep cannulation and contrast injection

Subsequently, sectioning of the muscle fibers and of the common bile duct mucosa was performed, with bile outflow in most cases. Once the common bile duct mucosa was identified, the guide wire was introduced, confirmed by radioscopy and bile duct contrast (Fig. 3) (video 1).

**Fig. 4:**
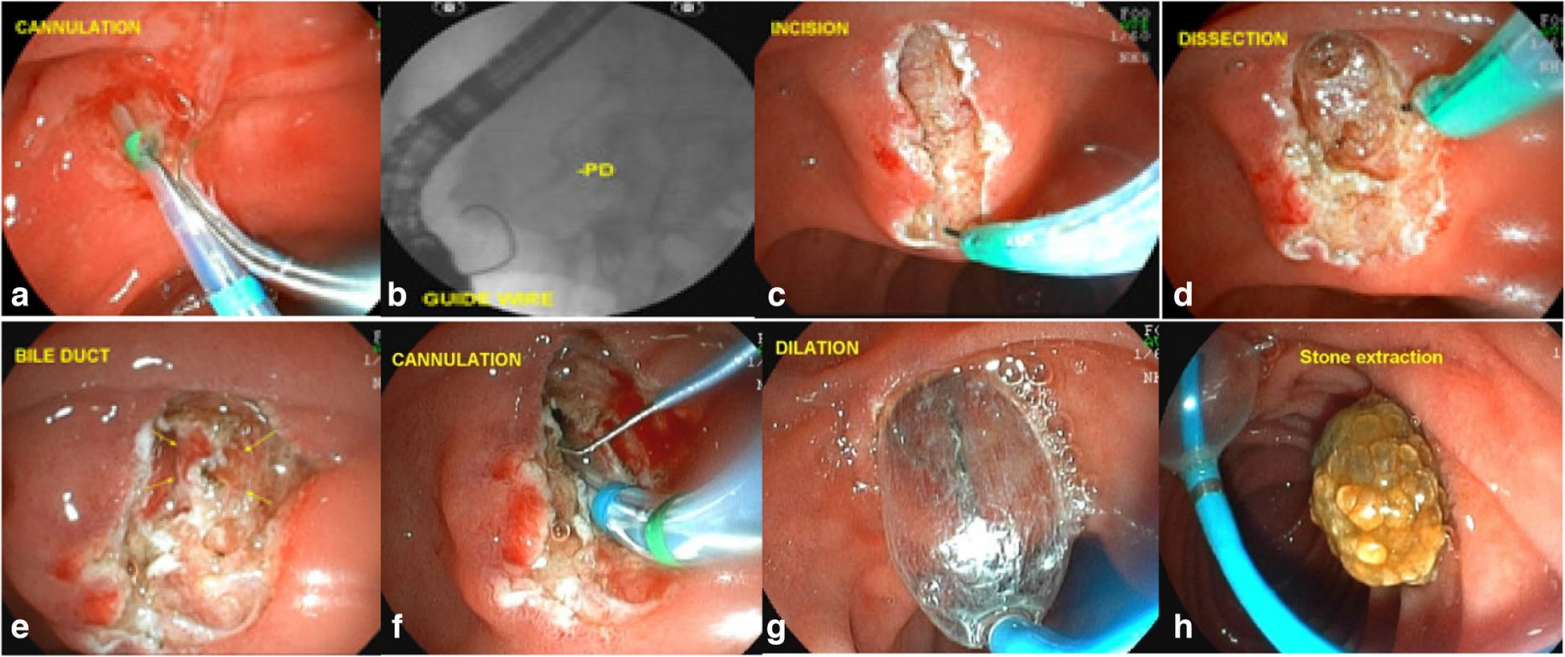
Enlarged fistulotomy of the papilla. **a** failed cannulation with the sphincterotome and guide wire; **b** failed cannulation with the guide wire and after injection of a small amount of contrast; **c** EFP with wide incisions; **d** dissection of the papilla; **e** identification of the muscular sphincter of the papilla, the muscular fibers, and the common bile duct mucosa; **f** easy cannulation of the bile duct after dissection; **g** dilation with 12 mm balloon of the fistula orifice; **h** common bile duct stone removal

If necessary, we can perform dilation with a 12- or 15-mm balloon catheter to facilitate removal of larger stones (Fig. 4).

The examinations were performed with the patients in the prone position, with noninvasive heart rate monitoring and pulse oximetry throughout the procedure and the use of routine supplemental oxygen. Intravenous medication was administered for sedation with midazolam, fentanyl and propofol, performed by an anesthesiologist. The devices used were a Fujinon® 4400 series duodenoscope (Fujifilm, Tokyo, Japan), sphincterotome, guide wires and needle-knife brands Olympus®, Boston®, Cook®, MediGlobe®, Scitech® or GFE®. None of the authors have any conflicts of interest.

### Postprocedurale care

Patients were contacted post-procedure by a member of the team or by the physicians responsible for the case. Complications such as bleeding, post-ERCP pancreatitis, and cannulation failure were discussed. Patients who presented abdominal pain, nausea and vomiting within 48 hours were examined by a surgeon and amylase and lipase tests were collected. Patients who had bleeding during the procedure or late bleeding were monitored and hematocrit and hemoglobin tests were ordered. Patients or family members were contacted 12, 24, and 48 hours after the procedure and instructed to contact their physician to report any ERCP-related symptoms. When necessary, patients were monitored for days or weeks, according to their clinical condition.

## Results

A total of 2233 exams were evaluated in 1959 patients, comprising 845 cases in males (37.84%) and 1388 cases in females (62.16%). The mean age was 56 years (Table 1).

**Table 1:** Diagnostic findings in ERCP

*Min. Age*	11	ERCP							
*Max. Age*	96	November 2006 to August 2022							
*Average*	56	No. of PATIENTS		1959					
*Median*	58	No. ERCP		2233					
*Mode*	67	MALE		FEMALE		TOTAL	(%)	M (%)	F (%)
		845	37.84%	1388	62.16%				
		DIAGNOSES							
Choledocholithiasis		434		823		1257	56.29%	34.53%	65.47%
Minimal changes		129		248		377	16.88%	34.22%	65.78%
Pancreatic head neoplasm		106		106		212	9.49%	50.00%	50.00%
Neoplasms of the hepatic hilum		42		67		109	4.88%	38.53%	61.47%
Benign fibrotic strictures		39		40		79	3.54%	49.37%	50.63%
Neoplasm of the papilla of Vater		46		38		84	3.76%	54.76%	45.24%
Mirizzi syndrome		6		15		21	0.94%	28.57%	71.43%
Postoperative complications		8		15		23	1.03%	34.78%	65.22%
Sclerosing cholangitis		13		7		20	0.90%	65.00%	35.00%
Chronic pancreatitis		10		1		11	0.49%	90.91%	9.09%
Other		12		28		40	1.79%	30.00%	70.00%

Direct cannulation of the bile duct with the sphincterotome was achieved in 76.36% of the 2233 cases, and 528 cases underwent EFP, with success in 465 cases (88.06%) and 63 failures (11.94%). Of the patients with failures, 33 (52.38%) returned for a second EFP attempt, with success in 30 cases (90.9%) and failure in 3 cases (9.1%). Deep bile duct cannulation was achieved in 93.75% of EFP procedures, and cannulation failure occurred in 33 cases (6.25%). Ultimately, bile duct cannulation was achieved in 98.52% of the 2233 cases and cannulation failure occurred in 33 cases (1.48%) (Table 2).

**Table 2:** Success and failure of ERCP procedures

Procedure	Success		Failure		Cases
	n (%)	CI 95%	n (%)	CI 95%	
CTPO	1705 (76.35%)	74.59–78.12	528 (23.65%)	21.88–25.41	1672
EFP1	465 (88.07%)	85.3–90.83	63 (11.93%)	9.17–14.7	528
EFP2	30 (90.91%)	81.1–100	3 (9.09%)	0–18.9	33
Final cannulation	2200 (98.52%)	98.02–99.02	33 (1.48%)	0.98–1.98	2233

*CTPO* cannulation through the papillary ostium; *EFP1* enlarged fistulotomy of the papilla first attempt; *EFP2 *enlarged fistulotomy of the papilla second attempt; *CI* confidence interval

There were 1760 procedures in the papilla (CP, EFP) and minor bleeding occurred during the procedure in 44 cases (2.50%): 10 cases by CP (0.56%) and 34 by EFP (1.93%) (Table 3).

**Table 3:** Minor bleeding during ERCP

Complications	Cases	CP		EFP	
		n (%)	CI 95%	n (%)	CI 95%
Bleeding in ERCP	44	10 (22.73%)	10.34–35.11	34 (77.27%)	64.89–89.66
Total	1760	10 (0.57%)	0.22–0.92	34 (1.93%)	1.29–2.57

*CP* conventional papillotomy; *EFP* enlarged fistulotomy of the papilla

Delayed minor bleeding, observed the day after the procedure, occurred in 23 cases (1.30% of the total), with 5 cases in CP (0.28%) and 18 cases in EFP (1.02%). Post-ERCP pancreatitis was observed in 8 cases (0.45%), all in the CP group (Table 4).

**Table 4:** Late complications of ERCP procedures

Post-ERCP complications	Cases	CTPO/CP		EFP1		EFP2	
		n (%)	CI 95%	n (%)	CI 95%	n (%)	IC 95%
Late bleeding	23	5 (21.74%)	4.88–38.6	18 (78.26%)	61.4–95.12	0	–
Post-ERCP pancreatitis	8	8 (100%)	–	0 (0%)	–	0	–

*CP* conventional papillotomy; *EFP1* enlarged fistulotomy of the papilla first attempt; *EFP2* enlarged fistulotomy of the papilla second attempt; *CI* confidence interval

## Discussion

Our approach to the papilla was always performed first with the sphincterotome and guide wire, avoiding trauma to the papilla and, if necessary, the injection of a small volume of contrast into the ostium to identify the anatomy of the junction of the pancreatic duct and the distal common bile duct. If the guidewire is directed only towards the pancreatic duct, we use the double-wire technique. If we still can’t cannulate the common bile duct, we move on to EFP. The choice between 0.025 or 0.035 wires does not appear to affect the success rate of selective cannulation or the incidence of pancreatitis [8]. However, the guide wire can cause damage to the pancreatic ducts, especially in multiple cannulation attempts. The guide wire, despite its flexibility, poses a risk of pancreatitis, which can cause intramural dissection, false trajectory and perforation of the main pancreatic duct or its secondary branches. The risk of pancreatitis ranges from 3.5 to 9.7%, and the mortality rate is 0.8% in cases of difficult cannulation [12, 13].

Difficult cannulation is a subjective term and can be challenging to define. ESGE published a guideline that considers one or more of the following situations difficult cannulation: more than five cannulation attempts; a duration of more than 5 minutes; and more than one cannulation or injection of contrast into the pancreatic duct [14]. The 2017 International Consensus, on the other hand, defined more than 10 minutes of duration or more than five attempts as difficult [3]. However, these guidelines are not widely accepted, and in daily practice, these rules are adapted to the different situations of each particular case. According to the results of a previously reported prospective study, the success rate of bile duct intubation in first-time papillary cases is 96% without precutting [15].

The macroscopic appearance of the major duodenal papilla influences the success of EFP, with larger and protruding papillae being easier to access, while small and intradiverticular papillae are more difficult to access [10]. These aspects were taken into account in the decision to start EFP earlier in cases of protruding and elongated papillae and greater persistence in the attempt to cannulate through the ostium in small and intradiverticular papillae, since dissection is more difficult in these cases.

Our approach when the guide wire does not enter the common bile duct within three or four attempts and does not enter the pancreatic duct is to initiate papilla dissection [16]. With EFP, cannulation of the bile duct is often easily achieved, and does not require extensive dissection. In a few cases, extensive dissection was necessary until access to the bile duct was achieved. Most endoscopists, who perform fistulotomies, find the duct with a single incision [17]. We prefer a wider opening of the mucosa and submucosa from the beginning of the procedure, because if bleeding occurs, the blood vessel is easily identified, allowing for more effective hemostasis. Another possible advantage of the wide exposure of the structures is the formation of a biliary fistula in the subsequent days, which facilitates a second attempt if success is not achieved in the first.

However, in 63 cases (EFP failure), the guidewire was not passed into the common bile duct, most of the time due to minor bleeding or edema and loss of identification of structures in the dissection area. These cases were considered failure of the EFP in the first attempt. Of these 63 patients, 33 returned for a second attempt and what we found was a biliary fistula, with an easily identified orifice allowing the passage of the guide wire and the sphincterotome. However, in 30 cases, the patients did not return for the second attempt for various reasons. The reasons for non return were, worsening of the biliary obstruction or the clinical condition of the patient, complications of the underlying disease, the decision of the surgeon who conducted the case, and the indication of other alternatives for biliary drainage. As a result, 30 EFP patients did not return for the second attempt. Most of these cases were advanced cancer of the head of the pancreas that did not allow the guide wire to pass through the tumor. Such patients were referred for endoscopic ultrasound-guided biliary drainage, percutaneous transhepatic drainage or surgery.

According to Flumignan [18] and Deng [19], a second fistulotomy attempt is feasible, and safe, and can be attempted after a short 48-hour interval if the first ERCP attempt is unsuccessful.

**Fig. 5:**
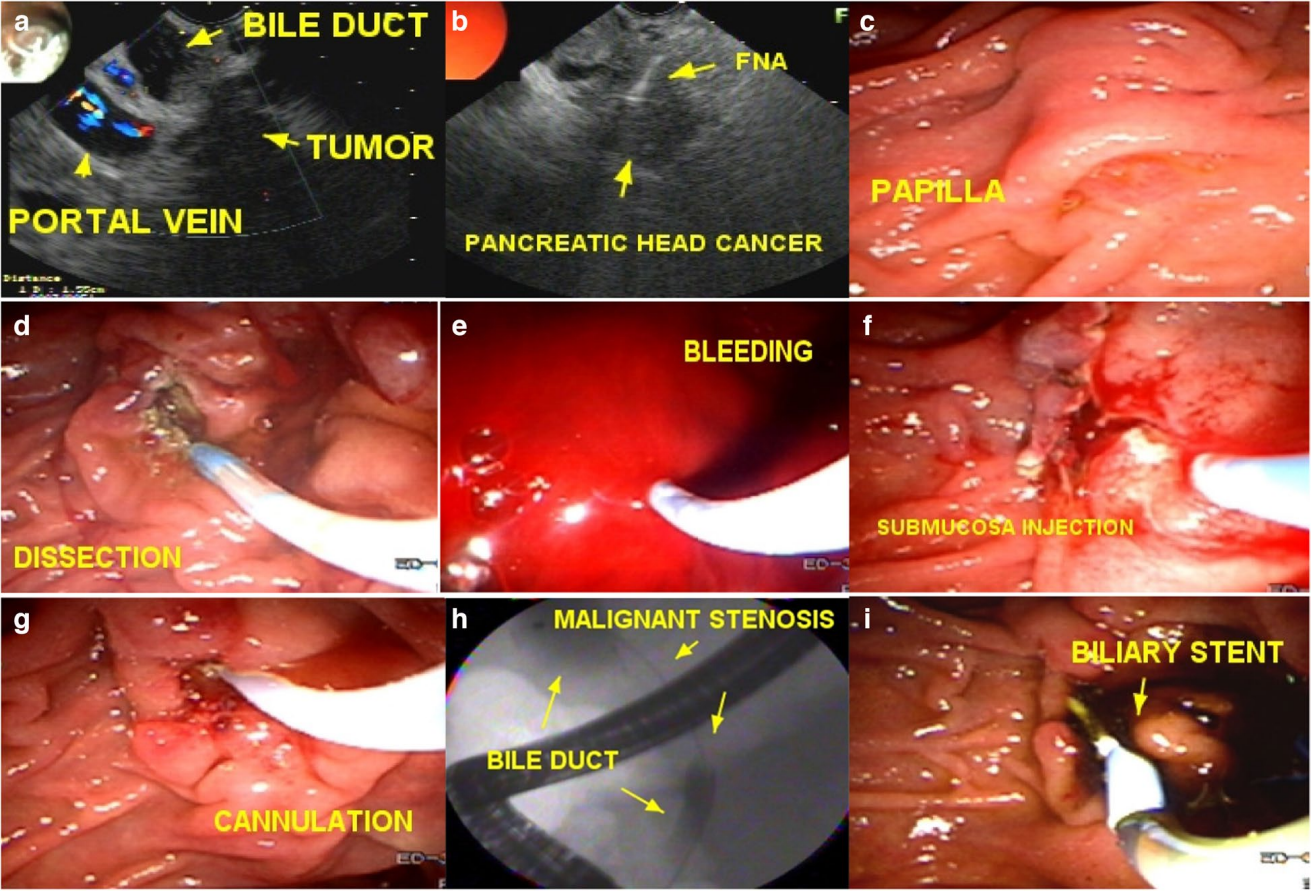
EFP2: enlarged fistulotomy of the papilla second attempt. **a** pancreatic head tumor; **b** FNA of pancreatic câncer; **c** difficult papilla and failed cannulation with the guide wire; **d** EFP1 with wide incisions and dissection of the papilla; **e** bleeding; **f** hemostasis was achieved by submucosa injection of saline and coagulation; **g** 2 days later the fistular orifice is identified and easy cannulation of the common bile duct is performed; **h** passage of the guidewire through the malignant stenosis; **i** biliary stenting

There were 1760 procedures in the papilla (CP, EFP) and minor bleeding occurred during the procedure in 44 cases (10 cases by papillotomy and 34 by EFP). These cases of bleedings were easily controlled by hemostatic measures using a submucosal injection of 25% glucose with 1:20000 adrenaline or hot biopsy forceps and bleeding point coagulation, and the procedure was completed or rescheduled for a new attempt. Those procedures rescheduled for a second time occurred from 2 hours later to 22 days (mean of 6.9 days) after the initial incision, with a median of approximately 6 days (Fig. 5).

Post-ERCP pancreatitis was seen in 8 cases, all in the CP group and none in the EFP group. Among the 8 patients in the papillotomy group, 5 had mild pancreatitis, and 3 had moderate pancreatitis. No perforation or other complications with CP or EFP occurred.

There were 23 cases of late bleeding, with 5 cases in CP and 18 cases in EFP. In these cases of late bleeding, which occurred the day after the procedure, the presence of melena and a drop in hematocrit were observed. However, none of these cases required blood transfusion or endoscopic hemostasis.

**Fig. 6:**
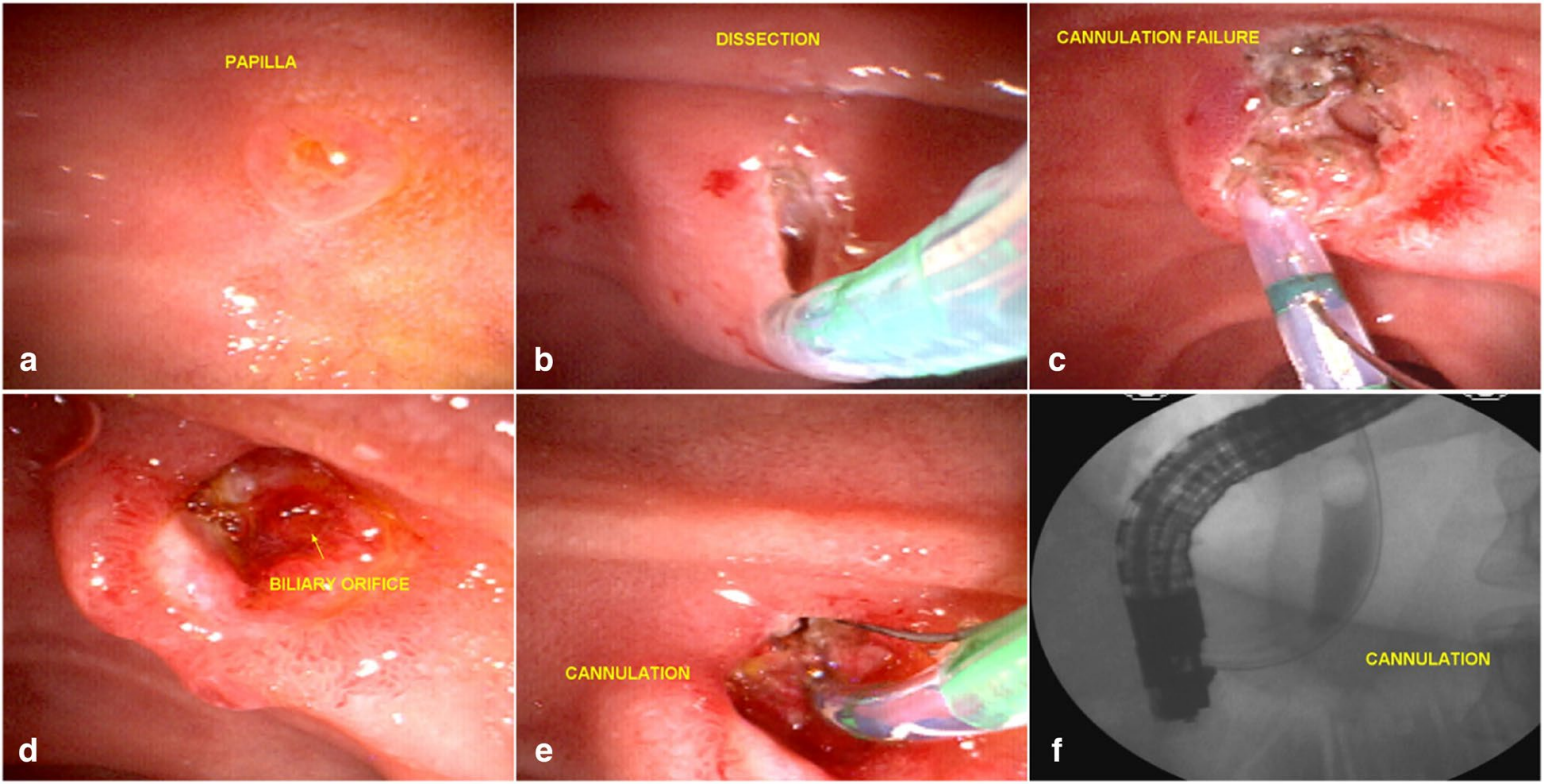
Failed EFP in the first attempt. **a** small papilla and failed cannulation with the sphincterotome and guide wire; **b** EFP with wide incisions; **c** failed cannulation after dissection; **d** 6 days later: the bile duct is easily visualized by staining the bile around the fistula orifice; **e** cannulation of the bile duct; **f** deep cannulation and contrast injection

This reinforces the idea that suprapapillary dissection is safer than countless unsuccessful attempts at cannulation through the ostium. The occurrence of acute post-ERCP pancreatitis is more dangerous than the sporadic occurrence of mild bleeding from papillary dissection, which is generally easily controlled. Another important point of the EFP procedure is that the wide exposure of the structures of the papilla leads to the formation of a fistula that, in most cases, is easily visualized by staining the bile around the fistular orifice, facilitating the cannulation of the bile duct (Fig. 6).

The 3 cases of failure in the second attempt at deep cannulation occurred in patients with advanced neoplasm of the head of the pancreas, in which the infiltration caused a major deformity of the common bile duct, making it impossible to pass the guide wire. These cases were referred for transhepatic transcutaneous biliary drainage or for endoscopic ultrasound-guided biliary drainage.

## Limitations

This was a retrospective study, evaluating intra- and postprocedural complications. We believe that a prospective randomized comparative study between CTPO and EFP performed directly, without prior attempts at cannulation with a guide wire, will be necessary. Another limitation is that the presente study was performed in a single center, albeit with professionals experienced in ERCP.

## Conclusion

This study detailed the papilla dissection technique, enlarging the exposure and identifying step by step the layers of the wall of the major papilla. EFP showed efficiency in rescuing cases of CTPO failure. It did not induce post-ERCP pancreatitis compared to CTPO/CP, but had higher rates of minor bleeding than CP (not clinically significant), with no cases of perforation or false tract. The procedure was also focused on rescuing cases of failed access by EFP, since the wide dissection facilitates the posterior approach by EFP in the second attempt. Ultimately, bile duct cannulation was achieved in 98.52% of the 2233 cases and cannulation failure occurred in 1.48%. EFP is a technique that is easily performed by endoscopists with experience in ERCP, being safe and effective, with low risk and few comorbidities.

### Supplementary Information

**Additional file 1.**

## Data Availability

All data generated or analyzed during this study are included in this published article.

## Abbreviations

ERCP: Endoscopic Retrograde Cholangiopancreatography
EFP: Enlarged Fistulotomy of the Papilla
CP: Conventional Papillotomy
CTPO: Cannulation Through the Papillary Ostium
EFP1: Enlarged Fistulotomy of the Papilla first attempt
EFP2: Enlarged Fistulotomy of the Papilla second attempt
CI: Confidence Interval
